# High RBM3 expression in prostate cancer independently predicts a reduced risk of biochemical recurrence and disease progression

**DOI:** 10.1186/1746-1596-6-91

**Published:** 2011-09-28

**Authors:** Liv Jonsson, Alexander Gaber, David Ulmert, Mathias Uhlén, Anders Bjartell, Karin Jirström

**Affiliations:** 1 Department of Clinical Sciences, Pathology, Lund University, Skåne University Hospital, 221 85 Lund, Sweden; 2 Department of Clinical Sciences, Division of Urological Cancers, Lund University, Skåne University Hospital, 205 02 Malmö, Sweden; 3 Department of Proteomics, AlbaNova University Center, Royal Institute of Technology, 106 91 Stockholm, Sweden; 4 Science for Life Laboratory, Royal Institute of Technology, 106 91 Stockholm, Sweden

**Keywords:** RBM3, immunohistochemistry, prognosis, prostate cancer

## Abstract

**Background:**

High expression of the RNA-binding protein RBM3 has previously been found to be associated with good prognosis in breast cancer, ovarian cancer, malignant melanoma and colorectal cancer. The aim of this study was to examine the prognostic impact of immunohistochemical RBM3 expression in prostate cancer.

**Findings:**

Immunohistochemical RBM3 expression was examined in a tissue microarray with malignant and benign prostatic specimens from 88 patients treated with radical prostatectomy for localized disease. While rarely expressed in benign prostate gland epithelium, RBM3 was found to be up-regulated in prostate intraepithelial neoplasia and present in various fractions and intensities in invasive prostate cancer. High nuclear RBM3 expression was significantly associated with a prolonged time to biochemical recurrence (BCR) (HR 0.56, 95% CI: 0.34-0.93, *p *= 0.024) and clinical progression (HR 0.09, 95% CI: 0.01-0.71, *p = *0.021). These associations remained significant in multivariate analysis, adjusted for preoperative PSA level in blood, pathological Gleason score and presence or absence of extracapsular extension, seminal vesicle invasion and positive surgical margin (HR 0.41, 95% CI: 0.19-0.89, *p *= 0.024 for BCR and HR 0.06, 95% CI: 0.01-0.50, *p = *0.009 for clinical progression).

**Conclusion:**

Our results demonstrate that high nuclear expression of RBM3 in prostate cancer is associated with a prolonged time to disease progression and, thus, a potential biomarker of favourable prognosis. The value of RBM3 for prognostication, treatment stratification and follow-up of prostate cancer patients should be further validated in larger studies.

## Findings

### Background and hypothesis

Prostate cancer is the leading cause of cancer death in men in economically developed countries [1] and there was an estimated 328 000 cases in Europe in 2008 which makes it the most common form of cancer in men [2]. For localized cancer, radical prostatectomy is the most common treatment and it has shown a benefit in cancer specific survival in comparison to watchful waiting [3]. Nevertheless, in some cases the cancer will recur with detectable prostate specific antigen (PSA) concentrations in blood, known as biochemical recurrence (BCR) [4]. Apart from PSA, no diagnostic or prognostic biomarkers have yet been incorporated into clinical protocols for management and risk stratification of prostate cancer patients despite extensive research efforts [5,6]. Thus, there is still a great need for new biomarkers to predict the course of the disease and differentiate indolent from life threatening cancer.

The RNA-binding motif protein 3, RBM3, is a glycine rich protein with an RNA recognition motif (RRM) capable of binding to both RNA and DNA and it is one of the earliest proteins synthesized in response to cold shock [7,8]. RBM3 is up-regulated in various types of human malignancies [9-11] and the role of RBM3 as a putative cancer biomarker was originally unravelled using an antibody-based discovery approach http://www.proteinatlas.org[12-14]. Since then, RBM3 protein expression, in particular its nuclear localization, has been demonstrated to be associated with a significantly improved survival in several cancer forms, e.g. breast cancer [11], ovarian cancer [15], malignant melanoma [16] and colorectal cancer (Hjelm et al, Proteomics Clinical Applications, in press). In ovarian cancer, RBM3 has also been demonstrated to be an independent factor of good prognosis at the gene expression level [15] and *in vitro *data point towards a link between RBM3 and cisplatin sensitivity in ovarian cancer cells [15], possibly by regulating several cellular processes involved in maintenance of DNA integrity [17].

In this study, we investigated the prognostic impact of RBM3 expression in prostate cancer by immunohistochemical (IHC) analysis of benign and malignant specimens from 88 patients treated with radical prostatectomy.

### Patients and methods

The patient cohort consists of 88 patients, between 48-74 years, treated with radical prostatectomy for localized prostate cancer at Skåne University Hospital, Malmö, from 1998 - 2003. Patient and tumour characteristics are shown in Additional File 1. Written consent was obtained from the patients and the local ethics committee at Lund University approved the study. Histopathological, clinical and follow-up data were obtained from the clinical- and pathology records. Vital status and cause of death was obtained from the Swedish Cause of Death Registry up until December 2006. The mean follow up period was 58.5 months after which three patients had died and 85 patients were alive. BCR was defined as PSA > 0.2 ng/ml [18] with a confirmatory level and clinical progression included BCR, death from prostate cancer and adjuvant radiation therapy. During follow up, 14 (15.9%) patients had BCR and 19 (21.6%) patients had clinical progression. Five (5.7%) patients received adjuvant radiotherapy and three (3.4%) patients received adjuvant hormonal therapy.

For tissue microarray (TMA) construction, areas representing cancer and normal prostate tissue were marked on haematoxylin and eosin stained slides, whereby normal tissue was sampled in a noncancerous zone away from the tumour, in most cases the transition zone. Two 1.00 mm cores from areas with invasive cancer and one 1.00 mm core representing benign prostate tissue were sampled from each case and mounted in a new recipient block using a manual arraying device (MTA-1 Beecher Instruments Inc., Sun Prairie, WI, USA). For immunohistochemical analysis, 4 μm TMA-sections were automatically pre-treated using the PT Link system and then stained in an Autostainer Plus (DAKO; Glostrup, Copenhagen, Denmark) with the mouse monoclonal anti-RBM3 antibody AAb030038; Atlas Antibodies AB, Stockholm, Sweden, diluted 1:5000. The specificity of the antibody has been validated previously [15]. RBM3 staining was evaluated by two independent observers (LJ and KJ) who were blinded to clinical and outcome data. The fraction of cells expressing RBM3 in the nucleus (NF) were denoted a score from 0 (0-1%), 1 (2-25%), 2 (26-50%), 3 (51-75%) and 4 (> 75%) and the intensity (NI) was scored as 0 (negative), 1 (weak), 2 (moderate) and 3 (strong). A combined nuclear score (NS) of NF × NI, was then constructed as previously described [15]. Cytoplasmic staining intensity was graded as 0 (no staining), 1 (weak staining) and 2 (strong staining).

### Statistical analysis

Spearman's rho and Chi-square tests were used for analysis of the correlation between RBM3 expression and relevant clinicopathological characteristics. Kaplan-Meier analysis and log rank test were used to illustrate differences in BCR free time and progression free survival (PFS) according to RBM3 expression. For survival analyses, RBM3 expression (intensity × fraction) was dichotomized into weak vs strong using classification and regression tree analysis (CRT). Cox regression proportional hazards modelling were used to estimate the impact of RBM3 expression on BCR (with RBM3 as a continuous variable) and PFS (with RBM3 as a dichotomized variable) in both uni- and multivariate analysis. All tests were two sided. A p-value of 0.05 was considered significant. All statistical analyses were performed using SPSS version 17 (SPSS Inc, Chicago, IL).

## Results

**Figure 1:**
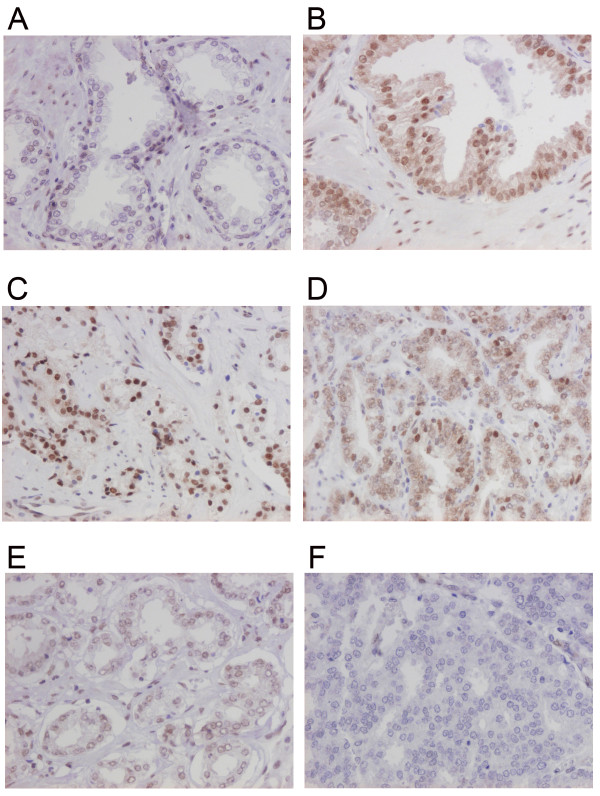
**RBM3 expression in normal prostatic epithelium, prostatic intraepithelial neoplasia and in prostate cancer**. Immunohistochemical images of RBM3 expression in (A) normal prostatic epithelium, (B) prostatic intraepithelial neoplasia and invasive prostate cancer, ranging from (C) high, (D) moderate, (E) weak to (F) negative expression.

**Figure 2:**
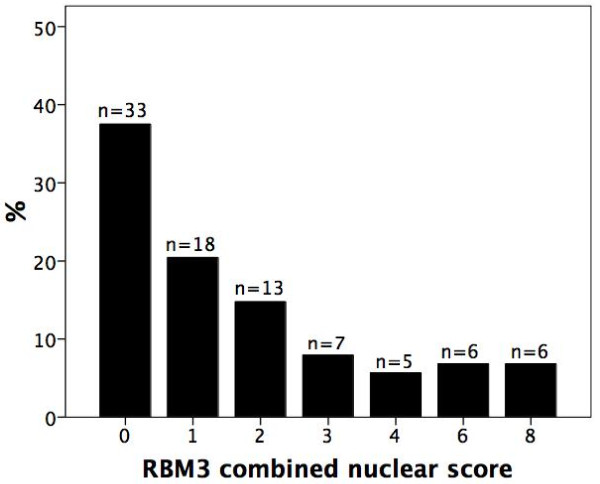
**RBM3 staining distribution in prostate cancer**. Distribution of nuclear RBM3 staining in invasive prostate cancer, denoted as nuclear score (fraction × intensity).

**Figure 3:**
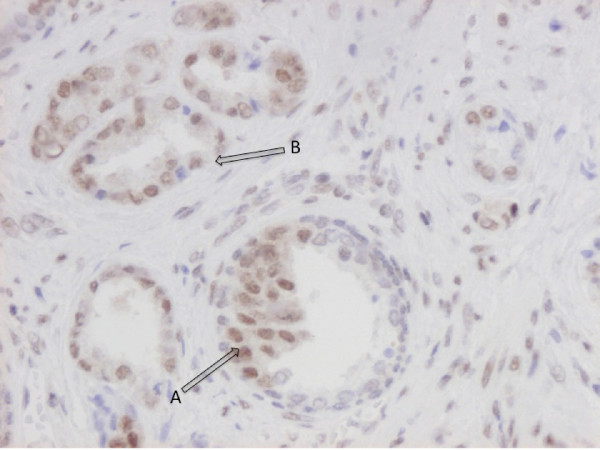
**Example of RBM3 up-regulation in prostate cancer progression**. Immunohistochemical image depicting up-regulated RBM3 expression in (A) PIN and (B) adjacent invasive component with high RBM3 expression.

While absent or weakly expressed in benign prostatic glands (Figure 1A), RBM3 was clearly up-regulated in prostatic intraepithelial neoplasia (PIN) (Figure 1B) and in invasive carcinoma RBM3 was expressed in various fractions and intensities (Figure 1C-E) with 33 (37.5%) cases lacking RBM3 expression (Figure 2). A progression sequence with up-regulated RBM3 expression in PIN and retained high RBM3 expression in the invasive component is illustrated in Figure 3. There was no obvious heterogeneity in the staining pattern between duplicate tissue cores.

**Figure 4:**
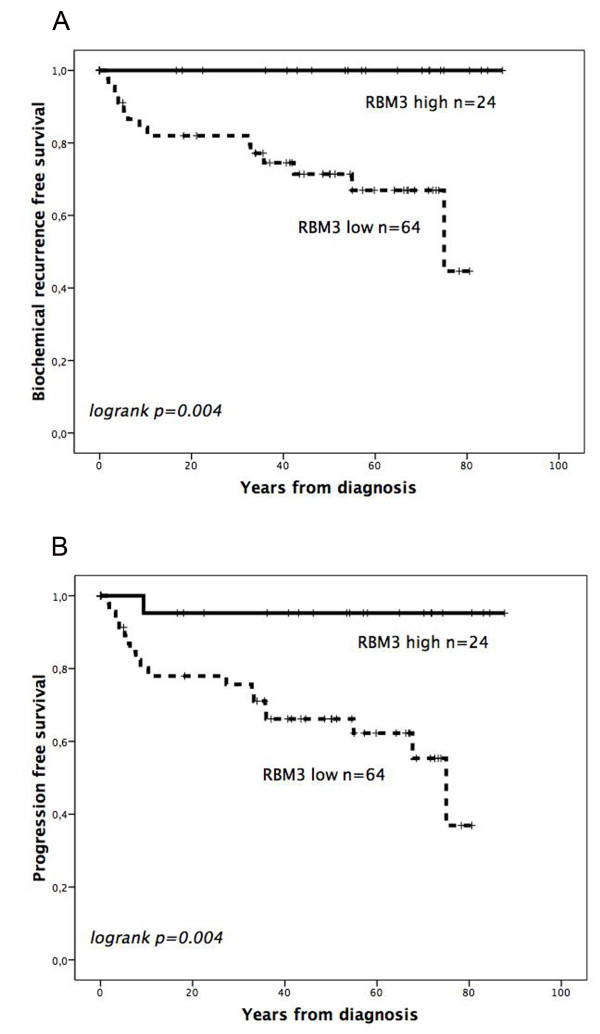
**Prognostic value of RBM3 expression in prostate cancer**. Kaplan Meier curves visualizing the impact of RBM3 expression on (A) biochemical recurrence free and (B) progression free survival.

CRT analysis suggested an optimal cutoff point at NS > 2 to determine the impact of RBM3 expression on BCR free survival and PFS. While there was no association between RBM3 expression and conventional clinicopathological parameters (Additional File 2), Kaplan Meier analysis demonstrated that high expression of RBM3 was associated with a significantly prolonged time to BCR (*p *= 0.004) and clinical progression (*p *= 0.004) (Figure 4). These associations were confirmed in Cox univariate analysis (HR 0.56, 95% CI: 0.34-0.93, *p *= 0.024 for BCR and HR 0.09, 95% CI: 0.01-0.71, *p = *0.021 for clinical progression) and remained significant in multivariate analysis, adjusted for preoperative PSA level, Gleason score and presence or absence of extracapsular extension, seminal vesicle invasion and positive surgical margins (HR 0.41, 95% CI: 0.19-0.89, *p *= 0.024 for BCR and HR 0.06, 95% CI: 0.01-0.50, *p = *0.009 for clinical progression) (Table 1). These significant associations were retained in both univariate and multivariate analysis also when the few patients who received adjuvant radiotherapy or hormonal therapy were excluded from the analysis (data not shown). Cytoplasmic RBM3 expression was not prognostic (data not shown).

**Table 1:** Cox univariate and multivariate analysis of biochemical recurrence free survival and progression free survival according to RBM3 expression

		Progression free survival	
RBM3 intensity*fraction dichotomized		HR (95% CI)	p-value
	n (events)		
Univariate			
Low (0-2)	64 (18)	1.00	
High (3-8)	24 (1)	0.09 (0.01-0.71)	0.021
			
Multivariate			
Low (0-2)	64 (16)	1.00	
High (3-8)	24 (1)	0.06 (0.01-0.50)	0.009
		**BCR free survival**	
RBM3 intensity*fraction continuous		**HR (95% CI)**	**p-value**
	n (events)		
Univariate	88 (14)	0.56 (0.34-0.93)	0.024
			
Multivariate	88 (12)	0.41 (0.19-0.89)	0.024

Multivariate analysis including adjustments for preoperative PSA level, Gleason score, presence or absence of extracapsular extension, seminal vesicle invasion and positive surgical margins.

### Interpretation and conclusions

Our results demonstrate that RBM3, while rarely expressed in normal prostatic gland epithelium, is up-regulated in PIN and invasive prostate cancer, and that patients with tumours expressing high nuclear levels of RMB3 have a significantly prolonged time to biochemical recurrence and clinical progression, also after adjustment for conventional prognostic factors. These findings are in line with previous findings in breast cancer [11], ovarian cancer [15], malignant melanoma [16] and colorectal cancer (Hjelm et al, Proteomics Clinical Applications, in press). Thus, there are increasing evidence for RBM3 being a biomarker of good prognosis in multiple cancer forms. The mechanisms behind these findings remain to be elucidated, an undertaking that might be somewhat challenging as current in vitro data point towards a proto-oncogenic role for RBM3 [8,9,19] and, hence, do not readily seem to support the clinical situation. However, the common hypothesis that oncogene activation is associated with an aggressive tumour phenotype does not always hold true, as well exemplified by the association between microsatellite instability and good prognosis in colorectal cancer [20]. It is evident that RBM3, while sparsely expressed in normal tissue, is up-regulated in most cancer forms and their pre-invasive stages *in vivo*. In light of the recently proposed association between RBM3 and DNA integrity in ovarian cancer [17], it could be speculated that RBM3 might play an important role in promoting early stages of tumourigenesis by interfering with the anti-cancer barrier provided by various DNA damage checkpoint mechanisms [21-23]. On the other hand, once a tumour has been established, an attenuated capability of DNA-damage response caused by RBM3 over expression might hinder the pressure for selection of more malignant clones [21,22]. Further studies are warranted to explore these associations.

In this study, we used a well-validated monoclonal antibody for detection of RBM3 expression [15,16]. Immunohistochemistry has several advantages compared to other assays in that it can easily be incorporated into clinical protocols and allows for assessment of the subcellular localization of proteins, which might have important prognostic implications. Our results indicate that immunohistochemical assessment of RBM3 expression in formalin-fixed paraffin embedded tumour samples could be a useful tool for prognostication and treatment stratification of prostate cancer patients. Since this was a small study these findings should be further validated in larger studies, preferably tumours from prospective, clinical trials.

## List of abbreviations

BCR: biochemical recurrence; CRT: classification regression tree analysis; DNA: deoxyribonucleic acid; HR: hazard ratio; IHC: immunohistochemistry; NF: nuclear fraction; NI: nuclear intensity; NS: nuclear score; PIN: prostatic intraepithelial neoplasia; PSA: prostate specific antigen; RBM3: RNA-binding motif 3; RNA: ribonucleic acid; RRM: RNA recognition motif; TMA: tissue microarray.

## Competing interests

The authors declare that they have no competing interests.

## Authors' contributions

LJ carried out the immunohistochemical analysis, performed the statistical analysis, and drafted the manuscript. AG assisted with the statistical analysis and helped draft the manuscript. DU and AB collected the clinical data. AB also contributed with clinical input. MU participated in the conception and design of the study. KJ participated in the conception and design of the study, the immunohistochemical analysis and helped draft the manuscript. All authors read and approved the final manuscript.

## Supplementary Material

Additional file 1 **Patient and tumour characteristics**. Click here for file

Additional file 2 **Association between RBM3 and clinicopathological parameters**. Click here for file
