# Supplementary material for: High RBM3 expression in prostate cancer independently predicts a reduced risk of biochemical recurrence and disease progression
Source: Diagn Pathol. 2011 Sep 28;6:91. doi: 10.1186/1746-1596-6-91 (PMC3195697; doi:10.1186/1746-1596-6-91)
Supplement: Additional file 1 — Patient and tumour characteristics. [file 1746-1596-6-91-S1.DOC]

| **Additional file 1. Patient and tumour characteristics** | |
| --- | --- |
|  |  |
| Median age at surgery (range) (yrs) | 63 (48-74) |
| Mean preoperative PSA (range) (ng/ml) | 9.6 (2.6-36.5) |
| Mean tumor volume (range) (ml) | 4.4 (0-14) |
| Clinical stage | n (%) |
| T1 | 40 (45.5%) |
| T2 | 47 (53.4%) |
| T3 | 1 (1.1%) |
| Gleason sum |  |
| <=6 | 48 (54.6%) |
| 7 | 35 (39.8%) |
| >=8 | 4 (5.6%) |
| Extracapsular extension |  |
| Yes | 53 (60.2%) |
| No | 30 (34.1%) |
| Missing | 5 (5.7%) |
| Seminal vesicle invasion |  |
| Yes | 13 (14.8%) |
| No | 75 (85.2%) |
| Positive surgical margins |  |
| Yes | 48 (54.5%) |
| No | 40 (45.5%) |
| Lymph node involvement |  |
| Yes | 2 (2.3%) |
| No | 33 (37.5%) |
| Missing | 53 (60.2%) |
|  |  |
