# Supplementary material for: High RBM3 expression in prostate cancer independently predicts a reduced risk of biochemical recurrence and disease progression
Source: Diagn Pathol. 2011 Sep 28;6:91. doi: 10.1186/1746-1596-6-91 (PMC3195697; doi:10.1186/1746-1596-6-91)
Supplement: Additional file 2 — Association between RBM3 and clinicopathological parameters. [file 1746-1596-6-91-S2.DOC]

| **Additional file 2. Association between RBM3** | | | |  |
| --- | --- | --- | --- | --- |
| **expression and clinocopathological parameters** | | | |  |
|  |  |  |  |  |
|  | **RBM3 expression** | |  |  |
|  | Low | High |  |  |
| n(%) | 64 (72.7) | 24 (27.3) | *p-value* |  |
|  |  |  |  |  |
| **Gleason sum** |  |  |  |  |
| 5 | 14 (21.9) | 4 (16.7) | 0.802 |  |
| 6 | 21 (32.8) | 9 (37.5) |  |  |
| >=7 | 29 (45.3) | 11 (45.8) |  |  |
|  |  |  |  |  |
| **Clinical stage** |  |  |  |  |
| T1 | 31 (48.4) | 9 (37.5) | 0.490 |  |
| T2 | 32 (50) | 15 (62.5) |  |  |
| T3 | 1 (1.6) | 0 (0) |  |  |
|  |  |  |  |  |
| **Tumour volume** | |  |  |  |
| 0-2 | 4 (15.4) | 6 (42.9) | 0.155 |  |
| 3-4 | 9 (34.6) | 3 (21.4) |  |  |
| 5-14 | 13 (50) | 5 (35.7) |  |  |
| *missing* | 38 | 10 |  |  |
|  |  |  |  |  |
| **Extracapsular extension** | |  |  |  |
| Yes | 38 (64.4) | 15 (62.5) | 0.872 |  |
| No | 21 (35.6) | 9 (37.5) |  |  |
| *missing* | 5 |  |  |  |
|  |  |  |  |  |
| **Seminal vesicle invasion** | |  |  |  |
| Yes | 10 (15.6) | 3 (12.5) | 0.717 |  |
| No | 54 (84.4) | 21 (87.5) |  |  |
|  |  |  |  |  |
| **Positive surgical margins** | |  |  |  |
| Yes | 37 (57.8) | 11 (45.8) | 0.320 |  |
| No | 27 (42.2) | 13 (54.2) |  |  |
|  |  |  |  |  |
| **WHO grade** |  |  |  |  |
| 1 | 6 (9.5) | 2 (8.3) | 0.282 |  |
| 2 | 37 (58.75) | 18 (75) |  |  |
| 3 | 20 (31.75) | 4 (16.7) |  |  |
| *missing* | 1 |  |  |  |
|  |  |  |  |  |
|  |  |  |  |  |
| RBM3 fraction*intensity Low = RBM3 <=2, High = RBM3 >=3 | | | | |
|  |  |  |  |  |
|  |  |  |  |  |
